# High rate and durable, binder free anode based on silicon loaded MoO_3_ nanoplatelets

**DOI:** 10.1038/srep10530

**Published:** 2015-05-22

**Authors:** Alejandro Martinez-Garcia, Arjun Kumar Thapa, Ruvini Dharmadasa, Tu Q. Nguyen, Jacek Jasinski, Theodore L. Druffel, Mahendra K Sunkara

**Affiliations:** 1 Conn Center for Renewable Energy Research; 2 Department of Chemical Engineering University of Louisville Louisville, KY 40292.

## Abstract

In order to make fast-charging batteries a reality for electric vehicles, durable, more energy dense and high-current density resistant anodes need to be developed. With such purpose, a low lithiation potential of 0.2 V vs. Li/Li^+^ for MoO_3_ nanoplatelet arrays is reported here for anodes in a lithium ion battery. The composite material here presented affords elevated charge capacity while at the same time withstands rapid cycling for longer periods of time. Li_2_MoO_4_ and Li_1.333_Mo_0.666_O_2_ were identified as the products of lithiation of pristine MoO_3_ nanoplatelets and silicon-decorated MoO_3_, respectively, accounting for lower than previously reported lithiation potentials. MoO_3_ nanoplatelet arrays were deposited using hot-wire chemical vapor deposition. Due to excellent voltage compatibility, composite lithium ion battery anodes comprising molybdenum oxide nanoplatelets decorated with silicon nanoparticles (0.3% by wt.) were prepared using an ultrasonic spray. Silicon decorated MoO_3_ nanoplatelets exhibited enhanced capacity of 1037 mAh g^−1^ with exceptional cyclablity when charged/discharged at high current densities of 10 A g^−1^.

Recent efforts have been focused on developing new materials to replace carbon anodes used in lithium ion battery technology for the improvement of energy density and durability. Specifically, nanostructured materials involving tin, silicon and their alloys with other transition metals are being extensively studied for their potential to enhance durability and attain higher capacities. In addition to silicon and tin based anodes, Molybdenum Oxide (MoO_3_) is also showing potential for durable anode material due to its ability to intercalate lithium ions into its layered structure without much chemical and mechanical degradation. However, the main challenges are the determination of correct material phase and adequate material configuration for achieving potentials below 0.7 V for Li intercalation. Typical MoO_3_ materials exhibit Li interaction potentials around 1.5 V vs. Li/Li^+^ leading to low energy capacities as anode materials.

The alpha phase of MoO_3_ presents an attractive layered crystalline structure with the (010) basal plane formed by double chains of edge-sharing [MoO_6_] octahedral connected through vertices and a reversible chemistry ideal for the task of repeatedly inserting and de-inserting Lithium ions^1^,^2^,^3^. Although MoO_3_ has a relatively low theoretical capacity of 1116 mAh g^−1^ compared to silicon, its structure allows it to be an insertion material for improved durability^4^. Previous studies from our group have shown that Mo_17_O_47_ nanowire arrays can retain a capacity of 630 mAh g^−1^ for up to 20 cycles at a current density of 50 mA g^−1^ 5. Also it has been reported that silicon coated MoO_3-x_ hybrid architectures, deposited by microwave plasma CVD and HFCVD respectively, can increase the storage capacity to 780 mAh g^−1^. However, due to the way the Si is deposited, the silicon layer was partially oxidized, thus potentially diminishing intercalation capacity, creating a barrier for lithium diffusion, and hampering electron transfer. Also, silicon deposition at elevated temperatures over few hundred degrees C and low pressures used in chemical vapor deposition allows for substantial oxygenation of deposited layers. Here, an ultrasonic spray is investigated for loading silicon nanoparticles onto MoO_3_ nanostructures, which could be made scalable for manufacturing in a roll-to-roll process.

In addition, the objective is to investigate the lithiation behavior of different phases of MoO_3_ nanostructures as well as the capacity and behavior of silicon loaded on MoO_3_ nanostructures. Also, it is necessary to understand the lithiation and de-lithiation mechanisms in silicon and MoO_3_ composites and the stability of these phases for durability. The composite anode presented here could pave the way for commercial lithium-ion batteries having higher energy storage capability and durability.

## Results

**Figure 1:**
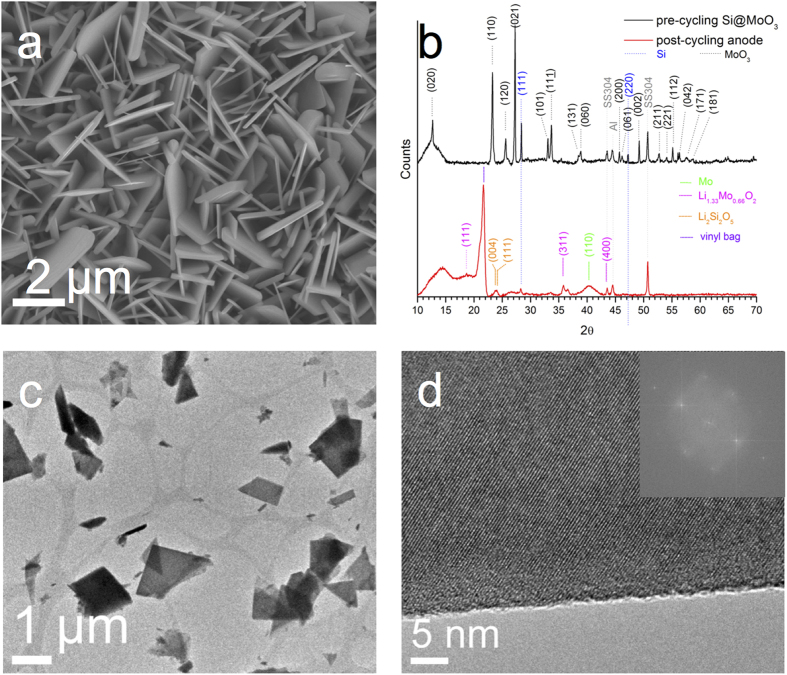
As-deposited MoO_3_ nanoplatelet arrays **a**) SEM (top view), **b**) XRD of Silicon loaded anode MoO_3_ [JCPDS 00–035–0609], Si [JCPDS 00–027–1402] before and after cycling Li_1.333_Mo_0.666_O_2_ [JCPDS 01–073–2300], Li_2_Si_2_O_5_ [JCPDS 00–015–0637] **c**) brightfield TEM of MoO_3_ platelets, **d**) HR-TEM of MoO_3_ platelet, inset FFT.

The hot-filament chemical vapor deposition (HFCVD) experiments using Mo filaments resulted in films that are opaque and light gray in colour. The deposited films were uniform over the entire substrate (1.5 cm diameter). Figure 1a shows that the films are composed of uniform arrays of nanoplatelets vertically aligned normal to the substrate. Similar morphology of thin nanoplatets have been reported in other studies^2^,^6^. The typical dimensions of each platelet are approximately 2 μm x 2 μm x 50 nm.

The diffraction pattern of the nanoplatelets shown in Fig. 1b is consistent with α-MoO_3_ (JCPDS 00–035–0609) having an orthorhombic crystal structure and cell parameters a=3.930 Å, b=13.8560 Å, c=3.6966 Å. Reflections from the underlying stainless steel substrate are observed at 2θ= 43.5 and 51 deg. The phase identification by XRD of the silicon-decorated MoO_3_ was challenging at low loadings. So, the MoO_3_ sample was ultrasonically sprayed with a higher loading of silicon (1:5 mass ratio of Si:MoO_3_) and then characterized by XRD before and after cycling. Figure 1b (black diffraction pattern), clearly shows the (111) and (220) silicon reflections [JCPDS 00–027–1402] before the sample was cycled as anode. HRTEM imaging (Fig. 1c) of this material shows that the platelets are single crystals (d-spacing of 0.37 nm as seen in Fig. 1d and inset with Fourier transform) growing in the shape of triangles, parallelograms or pentagons. The presence of silicon in the lightly loaded samples was confirmed by EDAX in TEM after cycling the anode (See Figure S.I in the Supplementary information document).

**Figure 2:**
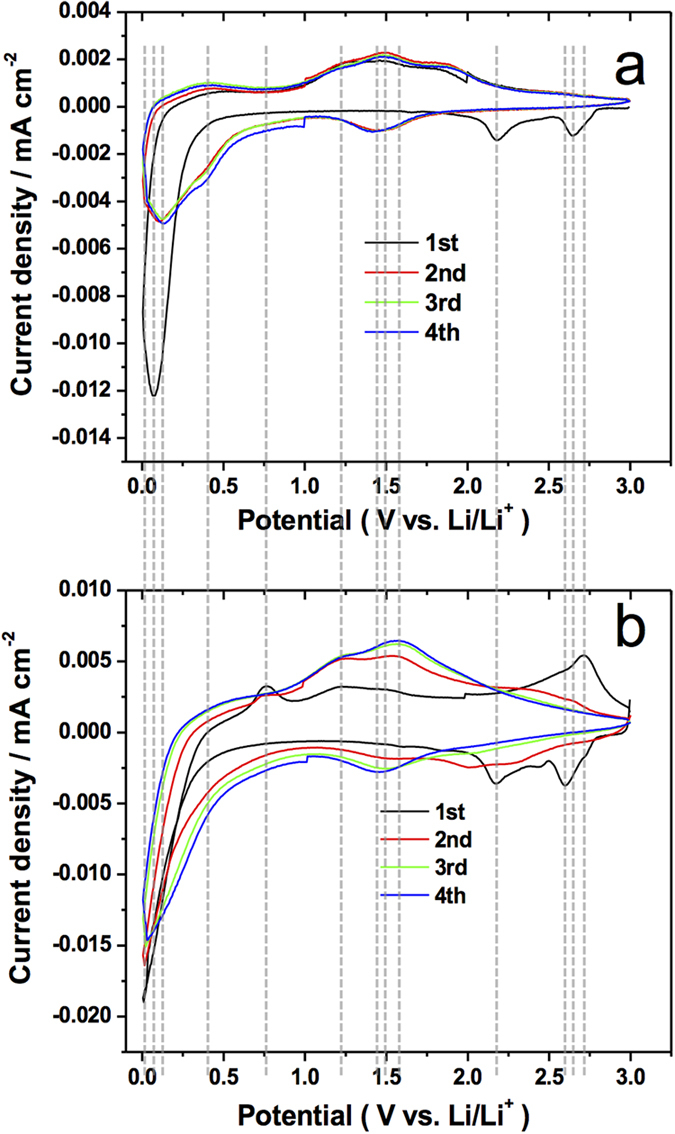
**a**) Cyclic voltammogram (CV) for as-deposited MoO_3_ nanoplatelet arrays, (MoO_3_ loading: 0.9 mg) (0.5 mg cm^−2^); **b**) Cyclic voltammogram (CV) for Silicon-sprayed MoO_3_ (MoO_3_ loading: 1.1 mg, Si loading: 25 sprays ~15 μg Si, ~1.3 wt% Si ) (MoO_3_ 0.625 mg cm^−2^, Si 8.5 μg cm^−2^).

For the electrochemical characterization, select set of anode samples were loaded with between 0.3 to 1.3 wt% of silicon on MoO_3_. Figure 2a and b show cyclic voltammograms obtained at a scan rate of 1 mV s^−1^ for as-synthesized MoO_3_ and Si@MoO_3_ respectively. In the pristine MoO_3_ anode (Fig. 2a), three cathodic waves are observed in the 3 V – 5 mV window for the first cathodic polarization sweep (discharging) at 2.65 V, 2.19 V and 75 mV. In the reverse scan (charging), the oxidation waves are seen around 0.5 V, 1.25 V and 1.5 V. Important evidence of irreversibility is observed in the subsequent cycles as the reduction waves over 2 V are not present anymore, and the cathodic peak centered at 75 mV appears with smaller current density than in the first cycle and shifts 50 mV to higher potentials. Interestingly, the second, third and fourth cycles are essentially identical and show no reactions at potentials over 2 V *vs*. Li/Li^+^. In Fig. 2a, the highest oxidation current is found at an Ep of 1.475 V for the 2^nd^ 3^rd^ and 4^th^ cycles.

In silicon-decorated MoO_3_ samples (Fig. 2b) three cathodic peaks were identified in the 3 V – 5 mV potential range at 2.6 V, 2.18 V and 20 mV during the first reduction process. Upon scanning back to 3 V in the first cycle, oxidation processes were observed at 0.75 V, 1.23 V, 1.5 V, and 2.72 V. Just like in the pristine MoO_3_ sample, the waves over 2.0 V disappear in the 3 and 4^th^ cycles. A new cathodic peak starts evolving at a potential of 1.45 V after the second polarization and its most obvious in the 4^th^ cycle.

Contrary to what happens in the as-synthesized electrode at low potentials, in the Si decorated electrode the magnitude of the cathodic peak at 20 mV was conserved to a great extent.

No evidence of plating was observed in the samples for the studied potential range.

**Figure 3:**
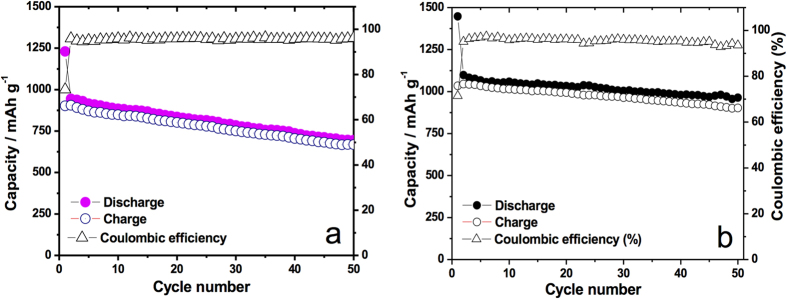
Specific capacity profiles at 100 mA g^−1^ for **a**) as-deposited MoO_3_ nanoplatelet arrays (MoO_3_ loading: 1.7 mg) (MoO_3_ 0.96 mg cm^−2^); **b**) Silicon-sprayed MoO_3_ (MoO_3_ loading: 0.6 mg, Si loading: 2 sprays ~1.2 μg Si, ~0.2 wt% Si) (MoO_3_ 0.34 mg cm^−2^, Si 0.7 μg cm^−2^).

**Figure 4:**
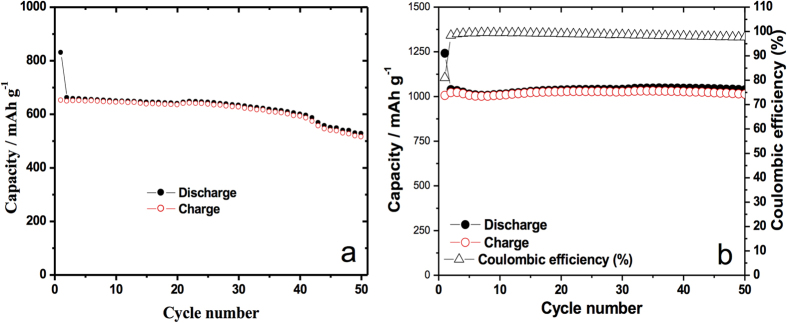
**a**) Specific capacity profiles at 10 A g^−1^ for MoO_3_ anode (MoO_3_ loading: 0.9 mg) (MoO_3_ 0.5 mg cm^−2^), **b**) Specific capacity profiles for silicon-sprayed MoO_3_ anode at 10 A g^−1^ (MoO_3_ loading: 0.4 mg, Si loading: 2 sprays ~1.2 μg Si, ~0.3 wt% Si). (MoO_3_ 0.23 mg cm^−2^, Si 0.7 μg cm^−2^).

Charge capacity versus cycle number data for both charging and discharging for the as-synthesized MoO_3_ and for Si@MoO_3_ is shown in Fig. 3a and b. The initial capacity for the pristine sample starts at around 1250 mAh g^−1^ and drops quickly in the second cycle to 950 mAh g^−1^, thereafter it slowly decays in a linear fashion to around 650 mAh g^−1^ after 50 cycles (at a cycling rate of 100 mA g^−1^). By contrast, the silicon decorated MoO_3_ (Fig. 3b) started at 1475 mAh g^−1^. In the second cycle it drops to about 1000 mAh g^−1^ and performed exceptionally well as it retained that capacity for over 50 cycles. Both pristine and silicon-decorated samples had coulombic efficiencies of over 97% in the studied range. More importantly, at a high current rating of 10 A g^−1^, charge capacity in the silicon sprayed MoO_3_ (Fig. 4b) continues to be above 1037 mAh g^−1^ after 50 cycles (0% steady state capacity fading), and has a coulombic efficiency of about 99%. This performance is substantially better than that of the pristine MoO_3_ in steady state, which started at 650 mAh g^−1^ and decayed to about 500 mAh g^−1^ after 50 cycles (23% capacity fading). The theoretical specific capacity of the composite anode, Qc, was calculated according to equation (1):
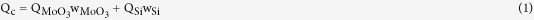


Where Q_MoO3_ and Q_Si_ are the theoretical capacities of pure MoO_3_ and pure silicon; w_MoO3_ is the mass fraction of MoO_3_.

The theoretical capacity of the composite anode having 0.3 wt% of silicon and 99.7 wt% of MoO_3_ is estimated to be 1130 mAh g^−1^. Therefore, the capacity retained after 50 cycles of 1037 mAh g^−1^ is noteworthy for this combination.

**Figure 5:**
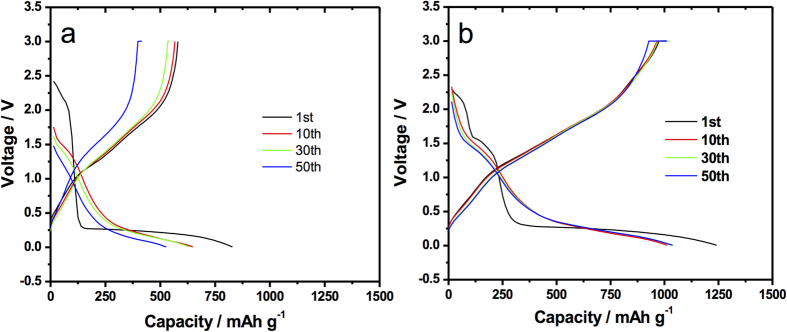
Charge/Discharge curves for **a**) as-synthesized MoO_3_ at 10 A g^−1^ (MoO_3_ loading: 0.9 mg) (MoO_3_ 0.5 mg cm^−2^), **b**) Si@MoO_3_ at 10 A g^−1^ (MoO_3_ loading: 0.4 mg, Si loading: 2 sprays ~1.2 μg Si, ~0.3 wt% Si) (MoO_3_ 0.23 mg cm^−2^, Si 0.7 μg cm^−2^).

Galvanostatic voltage profiles were recorded on charge/discharge for pristine and silicon-decorated MoO_3_ electrodes (Fig. 5) at 10 A g^−1^. During the first discharge cycle (Fig. 5a) no potential plateaus are observed above 1.5 V and only a sloping decay in voltage is observed to around 2.1 volts. According to several reports^7^,^8^, the features over 2 volts obey to intercalation of Li^+^ in between the lamellar MoO_6_ bilayers linked by weak van der Waals forces to form a solid solution of formula Li_0.25_MoO_3_ and are also linked to intercalation into the crystal lattice within the [MoO_6_] bilayers^2^. Next, the voltage decreases sharply to around 0.25 V. Multiple researchers have attributed the final potential drop to a conversion reaction leading only to Mo and Li_2_O, however in the present case, due to the rather low lithiation potential, this assertion is debatable at least particularly for intercalation into MoO_3_ nanoplatelets. X-ray diffraction data shown in Supplementary Figure S.II suggests that the final product, in the fully discharged state, after 100 lithiation cycles is rhombohedral Li_2_MoO_4_ (JCPDS 00–012–0763). The smoothness of the next charging curve and following cycles are characteristic of an amorphous Li_2_O transformation in the first discharge. In the second discharge cycle and from that point on, potential plateaus are observed at 1.5 V and from 0.25 to 0.18 V. In fact, such electrochemical features at potentials close to 150 mV could possibly be explained by the reactions shown in equations (2) and (3)
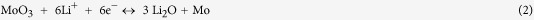

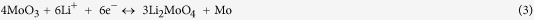


In the second discharge cycle and from then on, the reaction at 1.5 V has been associated with additional lithium uptake into the crystal lattice of MoO_3_^8^.

Other electrochemical features are observed in the Si@MoO_3_ sample tested at 10 A g^−1^. In this case, more pronounced potential plateaus are found at 2.2 and 1.5 V. The maximum capacity during the second cycle is around 1100 mAh g^−1^, which is greater than the capacity for the second cycle in the as-deposited MoO_3_. Likewise, in the samples tested at 10 A g^−1^, the presence of silicon particles on the MoO_3_ seems to have a notable effect in the stability of the anode since the 10st and 50th discharge profiles are essentially identical in Fig. 5b and the curves are much less spread than in Fig. 5a. At this high current density, the maximum capacities after the first discharge for the as-deposited MoO_3_ and the Si-sprayed MoO_3_ after the second cycle were found to be 650 and 1050 mAh g^−1^ respectively. In both cases presented in Fig. 5, the highest gain in capacity is obtained in the second lithiation stage under 0.25 V.

The charge-discharge curves at 100 mA g^−1^ are shown in Figures S.VIa and VIb for comparison. At this slower discharge rate the initial lithium intercalation is more obvious (Figure S.VIa) where well defined plateaus are present in the first cycle at 2.6 and 2.25 V vs. Li/Li^+^. Corresponding cathodic waves are clearly seen in the cyclic voltammetry plots of Figs. 2a and b at the same potentials observed in the charge/discharge curves.

**Figure 6:**
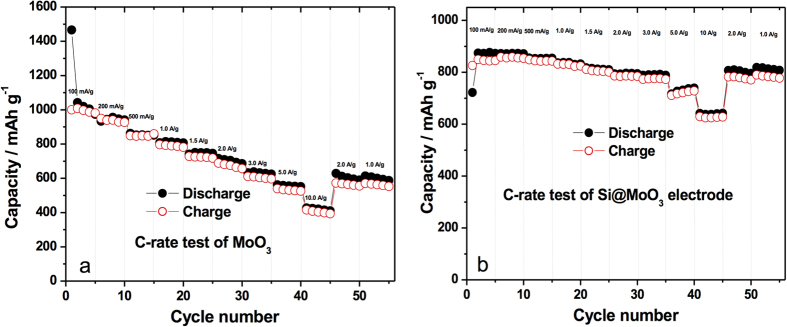
C-rate testing of **a**) as-synthesized MoO_3_ (0.9 mg of MoO_3_) (MoO_3_ 0.5 mg cm^−2^), **b**) Si@MoO_3_ (0.7 mg of MoO_3_, Silicon loading: 2 sprays ~1.2 μg Si, ~0.2 wt% Si) (MoO_3_ x mg cm^−2^, Si x μg cm^−2^).

C-rate tests for pristine MoO_3_ nanoplatelets and silicon-decorated MoO_3_ nanoplatelets are presented in Fig. 6a and b, respectively. From the plots it is obvious that the capacity of the as-synthesized MoO_3_ electrode faded faster than the silicon-decorated MoO_3_ electrode, as the current density was increased. The pristine MoO_3_ nanoplatelet sample of Fig. 6a presents 40% decay in capacity throughout the test after being cycled using increasing charge/discharge rates of 100, 200, 500, 1000, 1500, 2000, 3000, 5000 and 10000 mA g^−1^. By contrast the Si@MoO_3_ sample decayed only 27% when subjected to the same cycling conditions. The 2^nd^ cycle current density was used for calculating the percent decay. When the charge/discharge rate was reduced back to 2000 mA g^−1^ and 1000 mA g^−1^, the specific capacity recovered better in the Si decorated electrode.

**Figure 7:**
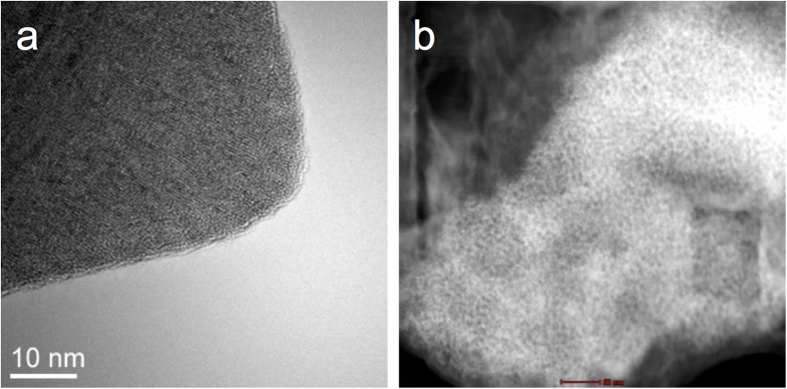
HRTEM **a**) anode before cycling, **b**) anode after lithiation/delithiation.

Morphological transformations have been observed by TEM in the lithiated samples as it is shown in Figs. 7a and b. Before lithiation the MoO_3_ platelets appear regular in shape and their facets are well defined. The d-spacing of the crystal is 0.37 nm. After lithiation/delithiation the anode adopts a sponge-like morphology with open pores and no apparent long-range periodicity, resembling an amorphous solid without facets as is obvious from Fig. 7b.

## Discussion

From the comparison between the diffraction patterns of a sample before and after cycling it is clear that some fraction of the α-MoO_3_ reduces to amorphous metallic molybdenum as can be deduced from the broad shape of the (110) diffraction peak of Mo. This conversion to molybdenum has been widely reported in the literature^5^,^7^,^9^,^10^. The red diffraction pattern in Fig. 2b, demonstrates that the main phase present in the cycled sample aside from Mo is Li_1.333_Mo_0.666_O_2_ [JCPDS 01–073–2300]. Additionally, the comparatively smaller intensity of the (111) reflection of silicon in the tested anode (vs. the original untested sample) tells us that silicon is converted to certain extent to some other compound. While Lithium intercalation into silicon has been extensively reported in the literature, we have found evidence of formation of lithium silicate with formula Li_2_Si_2_O_5_ [JCPDS 00–015–0637] which could possibly be a solid solution of 1 part of Li_2_O per 2 parts of SiO_2_^11^. Furthermore, Li_1.333_Mo_0.666_O_2_ is a different molybdate phase with high conductivity according to Electrochemical Impedance Spectroscopy data shown in Supplementary Figure S.III .

Equations (4) and (5) represent the electrochemical processes involved in the electrodes decorated with silicon.
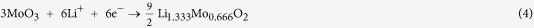

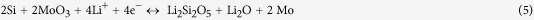


The voltammograms of the pristine MoO_3_ and Si@MoO_3_ (Figs. 2a and b) also depict the initial irreversibility observed in the discharge profiles of Fig. 5 and S.VI. Specifically, for the as-synthesized MoO_3_ electrode in the first sweep (black curve) two cathodic peaks are present at 2.65 and 2.18 V. These peaks have been interpreted in the literature as intercalation to form Li_0.2_MoO_3_ and Li_1.2_MoO_3_^12^. These features disappear in the subsequent cycles. One additional sign of an irreversible reaction is apparent at low potentials between 75 and 125 mV, where an initial high current density of -0.012 mA cm^−2^ is observed. In the later discharge polarizations the current drops to around half of the initial value. Interestingly, the second, third and fourth cycles are essentially identical and show no intercalation at potentials over 2 V vs. Li/Li^+^.

In the reverse sweep during cycle 1 of Fig. 2b an anodic peak is observed at 0.75 V. This feature is neither present after the second cycle nor in the pristine MoO_3_ voltammogram (Fig. 2a) so it necessarily corresponds to Si de-lithiation.

In the low potential range, after steady currents were attained in the 2^nd^ to 4^th^ cycles (Figs. 2a and b), the current peaks at two distinct values for pristine MoO_3_ nanoplatelets and Si-decorated MoO_3_ nanoplatelets, defining a ΔEp of -105 mV. This shift to lower potential between the non-decorated and Si-decorated anode material is evidence of the two different sets of reactions happening as it was described by equations 2, 3, 4, and 5. Such evidence suggests that MoO_3_ nanoplatelets lithiates to form Li_2_MoO_4_ while in the presence of Silicon, Li_2_Si_2_O_5_ and Li_1.333_Mo_0.666_O_2_ are produced. The Ep in both cases differ considerably from typical lithiation of bulk MoO_3_ that leads only to Mo and Li_2_O.

In Fig. 2b, the increase in current magnitude in the anodic peak at 1.65 V can be understood as follows: In the first discharge cycle there is lithium uptake to form a lithium molybdate phase, some of this lithium irreversibly stays in the anode and consequently the current at 1.65V is negligible in the first anodic polarization. In the next discharge cycle more Lithium is incorporated, which then de-intercalates as the voltage is swept back to more positive values (red anodic sweep). In the subsequent cycles this process is repeated until the current density stabilizes at around 0.006 mA cm^−2^.

Upon carefully inspecting the anodic peaks of Figs. 2a and b it was observed that in the Si-decorated anode, the peak with maximum current shifts by +75 mV with respect to the pristine MoO_3_ electrode.

In the state of the art materials, the typical potential range for incorporation of lithium and reduction to Li_2_O and Mo is reported to be around 0.4 to 0.5 V as summarized on Table 1 5,7,9,13,14. The cathodic wave at a potential as low as 0.125 V (Figs. 2a and b) has not been reported in the literature for MoO_3_ anodes, thus leading to believe that the products after discharge are the more conductive phases Li_2_MoO_4_ (JCPDS 00–012–0763) and Li_1.333_Mo_0.666_O_2_ (JCPDS 01–073–2300), rather than just Mo and Li_2_O. Due to its high band gap of 6 eV, Li_2_O has a poor electrical conductivity that limits the reversibility of conventional anodes by hampering charge transport^15^. Oxygen deficient phases afford increased p-type conductivity due to oxygen vacancies in the lattice^12^,^16^,^17^,^18^. Non stoichiometric phases, like Li(Li_1/3_Mo_2/3_O_2_), have been reported to have metallic conductivity^19^. A reduction process at such a low voltage between 125 mV and 250 mV is evident in both of the cyclic voltamogramms (Figs. 2a and b) and the charge/discharge curves (Figs. 5a and b, S.IVa and S.IVb).

Clearly, the majority of the capacity gain in the MoO_3_-based anodes of this study occurs at a potential of 0.2 V *vs*. Li/Li^+^ that is closer or below to the lithiation potential of silicon. As the lithiation potentials for MoO_3_ and Si are close, the MoO_3_ is a great host for silicon because a composite half-cell could operate at working voltages where MoO_3_ won’t get deeply discharged or irreversibly reduced to non-conductive Li_2_O and Mo. Additionally, with such characteristics the task of balancing the cathode, to engineer a safe full cell, is vastly simplified. Such a low lithiation potential plateau in the anode half cell, helps increase the open circuit potential of a working cell by maximizing the voltage difference between the positive and negative electrodes. The increase in observed capacity retention with silicon loading could not have come from silicon itself due to low loading involved (0.3 wt%). The silicon loading allowed for transformation of nanoplatelets into highly porous nanostructures. The high rate performance indicates that the resulting nanostructures after initial cycles of lithiation and de-lithiation maintain high conductivity. At low silicon loadings, the MoO_3_ nanoplatelet thin films perform closer to its theoretical capacity.

The role of MoO_3_, either as a cathode or anode for lithium ion batteries, has been the topic of intense controversy in the last decade because intercalation into MoO_3_ – and therefore a substantial fraction of its capacity – can occur at both high (>2 V) and low (<0.8 V) potentials^3^,^12^,^20^,^21^,^22^,^23^. Several authors have considered MoO_3_ as a potential cathode because it delivers a practical capacity of up to 300 mAh g^−1^, which seems high compared the capacities of the more conventional LiCoO_2_ and LiNiMnCoO_2_. However, the ability to intercalate lithium at high potential diminishes the usefulness of MoO_3_ as an anode, i.e. at low potentials.

The results of our investigation settle this controversy for MoO_3_ nanoplatelets as the low lithiation potential makes this nanostructured material more apt as a negative lithium ion battery electrode^5^.

HFCVD MoO_3_ nanoplatelets have shown improved cyclability performance and heightened capacity at extreme current densities with respect to other morphologies of this material like nanobelts with and without binders, nanoparticles and nanowires^5^,^7^,^9^,^13^,^24^. The anodes based on binderless nanoplatelets of the present study reach over 50 cycles with a capacity of 1000 mAh g^−1^ when discharged at a current density of 10 A g^−1^.

Based on the results of this work, the vertically aligned nanoplatelet structures seem to perform better than the other previously reported-nanostructures for lithium ion batteries. This is because the interconnected arrays of vertical MoO_3_ nanoplatelets, shown in Fig. 2a, hold the structure together preventing the morphology from collapsing after lithiation, and defines a backbone for metallic Mo and Li_2_O to further form Li_2_MoO_4_ and cycle back during delithiation in the charging process (see Supplementary Figures. S.II and S.IV). At low voltages close to 5 mV the crystal structure is partially destroyed as metallic molybdenum is formed on the surface but the general platelet-like shape remains as demonstrated by the electron micrographs presented in Figure S.IV . This characteristic makes vertically aligned MoO_3_ nanoplatelet -based electrodes more robust compared to other morphologies of MoO_3_ such as nanowires, while at the same time favors transport of lithium ions from the electrolyte to lithiation sites in the solid due to the large surface area. Nanoplatelets or nanoflakes of transition metal oxides with large interlayer spacings have shown potential for energy storage applications as they permit fast ion diffusion^25^.

Furthermore, the mechanism of lithium intercalation in layered MoO_3_ has been described as a process that begins with lithium adsorption on surface sites of low energy. The morphology of the sheets, shown in detail in Figure S.V, with an abundance of step and screw dislocations on their surface is unique to nanoplatelets of α-MoO_3_. These nanometer thick step edges are favored sites for lithium absorption where episodical intrasite jumps in the van der Waals intralayer regions lead to higher Li mobility^19^. Subsequently the displacement, reaction and creation of dislocations ease the insertion of lithium cations into the interior of the nanoplatelet^26^. Additionally, as lithium intercalates, strain can easily be relaxed by propagating dislocations on the surface of the platelets, which could help explain the extended durability of MoO_3_ nanoplatelets especially in the initial lithiation stages.

Core-shell hybrid architectures comprising MoO_3_ and SnO_2_, have been reported with exceptionally high capacities on the order of 2000 mAh g^−1^ for up to 30 cycles at a current density of approximately 100 mA g^−1^ 10. However, the presence of SnO_2_ in said anodes does not seem to affect its unfavorable capacity retention and stability upon cyclic even at such a low current density. By contrast, our silicon- sprayed MoO_3_ performs extraordinarily well despite the huge current density of 10 A g^−1^, as seen in Fig. 5b where the charge/discharge profiles do not change at all between cycles 1 and 50. In this case, a minute amount of silicon is responsible for this dramatic improvement in stability as is obvious by comparing Fig. 5a and b.

Recently, mesoporous orthorhombic MoO_3_ nanowire bundles were shown to exhibit an enhanced electrochemical performance at low current densities compared to nanobelts after a topotactic chemical transformation under vacuum^27^. However, the highest current density employed during testing was 1 A g^−1^ and the specific capacity decayed dramatically during the first 20 cycles and hardly retained 400 mAh g^−1^ at 50 cycles. Serious capacity fading with structure degradation, particularly at higher rates, has been a long-standing problem for transition metal oxides including MoO_3_ 28,29. We report here MoO_3_ anodes decorated with Si nanoparticles at a loading less than 0.3 wt% that retain over 1037 mAh g^−1^ when charged/discharged 50 times in very demanding current density conditions of 10 A g^−1^.

Attempts with three silicon loading concentrations were compared, namely 0.3, 1.5 and 4 wt% Si. However, no linear relationship between the capacity and amount of silicon was identified. The electrodes with 1.5 wt% and 4 wt% silicon were not decorated uniformly but agglomeration of particles occurred, possibly creating contact issues in the electrode. Given the large charge capacity of silicon and in view of its natural tendency to fade over time, anode performance could be further improved by optimizing the silicon loading concentration on MoO_3_.

Ultrasonic spraying of silicon nanoparticle suspensions proved to be an easy and convenient method for decorating MoO_3_ anodes. At low loadings of silicon, the MoO_3_ nanoplatelet arrays exhibited superior electrochemical performance, close to theoretical capacity, and excellent reversibility. The MoO_3_ nanoplatelets of this study showed a lithiation potential between 0.125 and 0.25 V vs. Li/Li^+^, with the highest capacity gain obtained at that low lithiation potential range. Electrochemical and diffraction data suggest that such low potentials obey to the formation of Li_2_MoO_4_ when nanoplatelets are lithiated, as well as to a transformation to Li_1.333_Mo_0.666_O_2_ when the metal oxide anode is decorated with silicon. Therefore, the MoO_3_ nanoplatelets have an ideal compatibility with silicon, and could very well replace graphite as a silicon host. At low loadings, silicon alloyed with MoO_3_ and formed nanostructured phases which were determined to have improved the reversibility of MoO_3_ in the lithiation and delithiation process, yielding longer-lasting anodes and enhanced capacity.

## Methods

Round stainless steel substrates 1.5 cm in diameter were cleaned by ultrasonication in ethanol/acetone for 15 minutes. The mass of the clean substrates was measured using an analytical scale. MoO_3_ coatings were deposited by hot filament CVD for 30 minutes in 100% oxygen at a flow rate of 10 sccm and a total pressure of 2.5 torr. A bias of 17 volts AC was applied to the molybdenum filament (0.5 mm in diameter and 6 ft in length) resistively heating it to temperature of about 800 °C. Further details of the experimental setup used for these deposition experiments are given in previous publications^5^,^30^,^31^. The mass of the MoO_3_ deposit in mg was calculated by subtracting the initial mass of the substrate from the final mass of each sample.

The morphology of these molybdenum oxide deposits were characterized by scanning electron microscopy (FEI Nova 600) in the secondary electron mode and high-resolution transmission electron microscopy (HRTEM) (Tecnai F20 FEI TEM at 200kV). Phase identification was performed by X-ray diffraction in the locked couple mode (Bruker D8 Discovery, Cu Kα).

**Figure 8:**
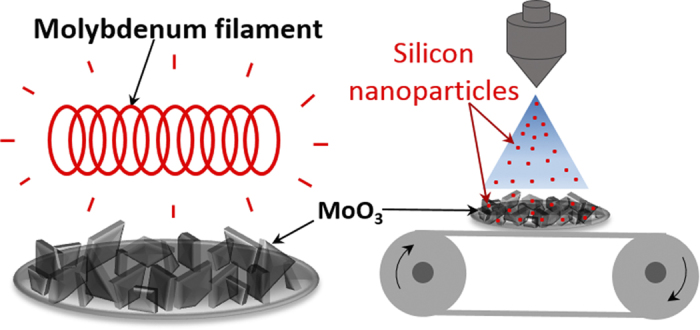
Processing Schematic: HFCVD of MoO_3_ nanoplatelets followed by ultrasonic spraying of silicon nanoparticle suspension onto a substrate mounted on a roll-to-roll process.

Select MoO_3_ deposits obtained by HFCVD were decorated with silicon nanoparticles by ultrasonic spraying of a silicon nanoparticle suspension in isopropanol/ethanol. This suspension was prepared by ball milling a silicon wafer according to the grinding method described by D. Reeves^32^. The silicon concentration was 0.8 mg ml^−1^ of suspension. The solutions were sprayed using a 48 kHz ultrasonic nozzle attached to Wide Track Coating system (SonoTek). The samples were placed on a hot plate heated to 120°C for approximately 2 minutes before being sprayed with the dispersion. In order to prevent large silicon aggregates being deposited on the samples, the dispersion was fed through a Hielscher UP400S Ultrasonic Processor prior to passing through the nozzle. A flow rate of 3.0 ml min^−1^ ensured a good spray pattern. A schematic of the electrode fabrication process is presented in Fig. 8. The ultrasonic spray uses a piezoelectric transducer to convert electricity to a high frequency signal, which then creates a standing wave at the surface of the nozzle. The vibrations break up the top of the wave, in to very small droplets. The 48 kHz nozzle has been rated to produce droplets with a mean diameter of 38 μm with water. If the particle size is smaller than the droplets, the droplets will encase one or more particles depending on particle size, agglomeration and concentration.

The amount of silicon sprayed on each substrate was estimated based on the known concentration of silicon in the spraying suspension. The spray flow rate, total spray area, substrate speed, and number of passes are all known experimental parameters that can be used to accurately calculate the silicon loading. The silicon loading on the MoO_3_ substrates was controlled by varying the number of times the samples passed below the nozzle. The mass of silicon sprayed on each substrate was determined according to the following equation (6):
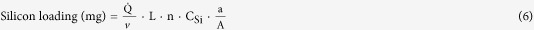


Where:


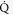
: suspension spray rate, (ml min^−1^)

*v* : belt speed, (cm min^−1^)

L : belt displacement, (cm)

n : number of passes of spray over substrate, non-dimensional

C_Si_: Silicon concentration in suspension, (mg ml^−1^)

a : electrode area, (cm^2^)

A : sprayed area of belt, (cm^2^)

To confirm the silicon loading estimation of equation (6), copper foil substrates (1.5 cm in diameter) were sprayed with the same silicon nanoparticle suspension used to prepare the silicon decorated electrodes. The mass of each substrate was registered with a microbalance before and after the spraying process. Different silicon loading levels were applied by increasing the number passes under the ultrasonic spray nozzle. A calibration curve is shown in Figure S. IXd. The silicon loading obtained with one single pass under the spraying nozzle was determined to be, by gravimetric method, around 0.3 μg. Due to the foregoing, we define the lower and upper limits for silicon mass loading on the electrodes between 0.3 and 1 μg per spraying pass. An average value of 0.6 μg per pass will be used for reporting the Si wt% in the decorated MoO_3_ samples.

Based on the mass of silicon and MoO_3_ the fraction of silicon is determined. For electrochemical characterization of the anodes different Si loading were studied in the range 0.7 to 13.6 μg cm^−2^, while the typical MoO_3_ loading density on the samples was on the order of 230 to 400 μg cm^−2^, equivalent to 0.3–6 wt% of silicon. The actual loading values and loading density for each tested sample are reported in the figure legends.

CR2032 coin-type cells were assembled in a glove-box under a dry inert atmosphere using MoO_3_ or Si@MoO_3_ as working anode and lithium foil as counter electrode separated with glass fiber filter (Advantec GB-100R, Toyo Rishi CO., Japan) saturated with 1M LiPF_6_-ethylene carbonate (EC) : dimethyl carbonate (DMC) (1:2 v/v). A 16-channel battery tester (Arbin Instruments, USA) was employed to carry out the charge-discharge measurements.

## Author Contributions

M.K.S and A. M conceived the experiments, wrote the manuscript and supplementary information text. A.M, A.K.T, and T.Q.N. prepared Fig. 1, 2, 3, 4, 5, 6, 7, 8 and Supplementary Figures S.I-S.VII. A.M, A.K.T. and J.J. performed synthesis, electrochemical and structural characterization of the samples. R.D. and T.D. performed the silicon loading by ultrasonic spray. All authors participated on the discussions, reviewed and commented on the manuscript.

## Additional Information

**How to cite this article**: Martinez-Garcia, A. *et al.* High rate and durable, binder free anode based on silicon loaded MoO_3_ nanoplatelets. *Sci. Rep.* **5**, 10530; doi: 10.1038/srep10530 (2015).

## Supplementary Material

Supporting Information

## Figures and Tables

**Table 1:** State of the art of lithium ion battery anodes based on MoO_3_

Material /Morphology	E (high)^a^ [V]	E (low)^b^ [V]	Binder	Capacity [mAh g^−1^]	Current density [mA g^−1^]	Cycles	Ref./Year
MoO_3-x_ nanowires	1.5	0.5 to 0.7	None	630	50	20	5 2012
Ultralong MoO_3_ nanobelts	2.6–2.2, 1.5	0.5 to 0.3	Na-CMC	730	200	200	6 2012
MoO_3_ nanobelts	2.75 and 2.25	0.5 to 0.4	None	400	2000	80	8 2013
MoO_3_ nanobelts	2.3 and 1.4	0.5 to 0.3	None	1067	558	50	11 2011
MoO_3-y_ powder	2.snd 2.3	0.5 to 0.3	Acetylene Black/PVDF	630	38	35	11 2009
MoO_3-x_ NW’s bundles	2.25	0.5 to 0.4	Acetylene Black/PVDF	490.5	1000	100	18 2014

a) High Potential Region ; b) Low Potential Region.
