# Supplementary material for: High rate and durable, binder free anode based on silicon loaded MoO3 nanoplatelets
Source: Sci Rep. 2015 May 22;5:10530. doi: 10.1038/srep10530 (PMC4441197; doi:10.1038/srep10530)
Supplement: Supporting Information [file srep10530-s1.pdf]

Supplementary Information

High Rate and Durable, Binder Free Anode Based on Silicon Loaded MoO_3 Nanoplatelets

Alejandro Martinez-Garcia, Arjun Kumar Thapa ,Ruvini Dharmadasa, , Tu Q. Nguyen, Jacek Jasinski, Theodore L. Druffel & Mahendra K Sunkara*

A lithium battery anode comprising α-MoO_3 and 0.3%w of silicon was disassembled in a glove box under inert atmosphere after 50 cycles of charge/discharge and a small sample was scraped off of the anode using a copper TEM grid. The sample was analyzed by EDAX in the High-resolution imaging mode of the transmission electron microscope. The results are presented in Figure S.I. The spectrum for selected area 1 shows a silicon Kα line at 1.739 keV, thus confirming the presence of silicon particles in the anode. Both regions 1 and 2 present Mo Kα line at energy of 17.441 keV.

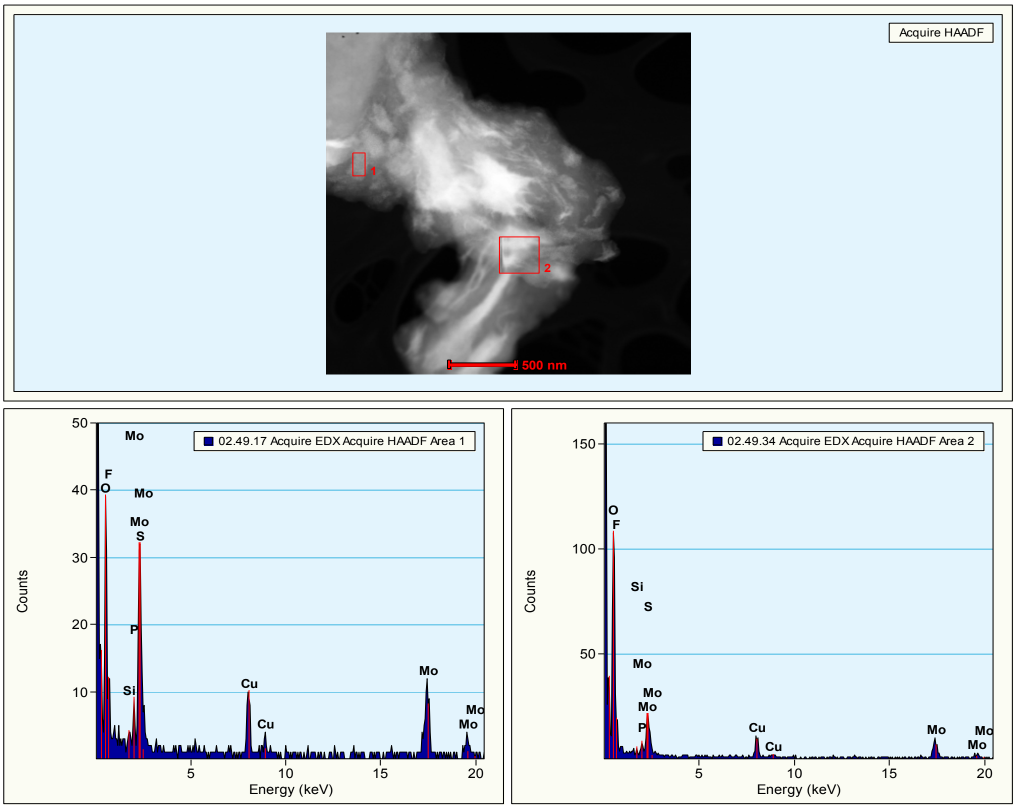

Figure S.I. Silicon decorated MoO_3 nanoplatelet after 50 lithiation/delithiation cycles. a) Darkfield TEM image, b) EDX spectrum of selected area 1 c) EDX spectrum of selected area 2.

X-ray diffractometry was performed on a tested anode in the discharged state after 100 cycles of lithiation/deliathiation. The results are shown in Figure S.II.

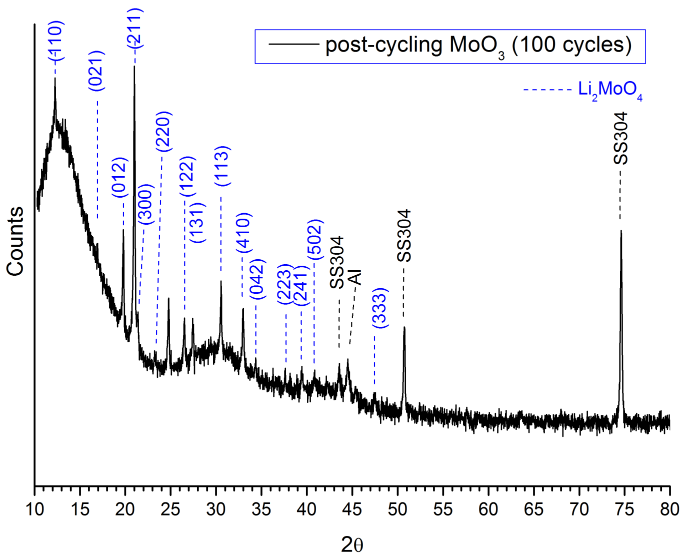

Fig S.II X-ray diffraction pattern for lithiated MoO_3 nanoplatelets in discharged state after 100 cycles. Li_2MoO_4 (JCPDS 00-012-0763)

Electrochemical impedance spectroscopy was used to assess the conductivity of samples after being tested for 15 cycles. The Nyquist plot shown in Figure S.III was obtained in the fully discharged state of the batteries and at 0 V vs. OCP.

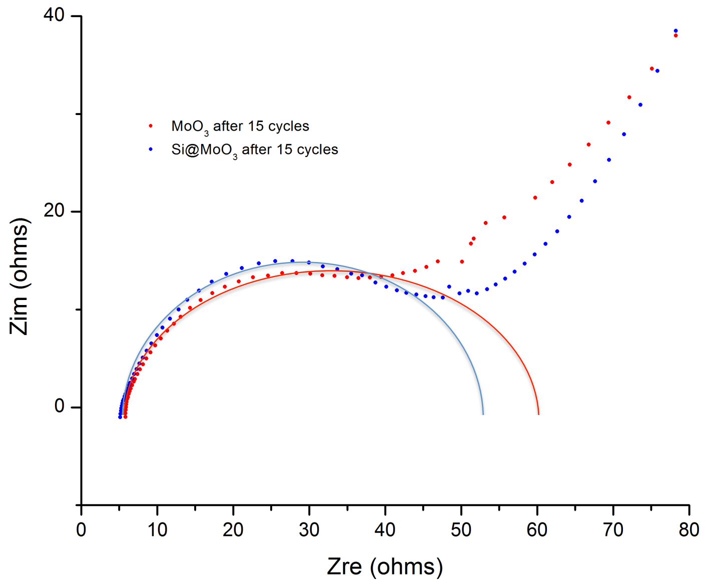

Fig S.III Nyquist Plot of pristine MoO_3 and Si-decorated MoO_3

The morphological transformation of the active lithiation material MoO_3 was assessed by depositing nanoplatelets on stainless steel substrates and cycling a coin cell battery for 100 times. The initial morphology is presented in Figure S.IVa, while the lithiated material is observed in figures S.IVb and S.IVc. The images clearly show that before lithiation the metal oxide nanoplatelets are all inter-connected with neighboring structures forming a network of nanoplatelets. In the discharged state (after lithiation), a few nanoplatelets are still visible as shown by the red outlines in Figures IVc and IVd. The general morphology of polygonal nanosized and interconnected sheets is preserved to a great extent, but porous and polycystalline layer covers the nanoplatelets. According to the results of the study the polycrystalline layer is a mixture of Li_2MoO_4, metallic molybdenum and Li_2O.

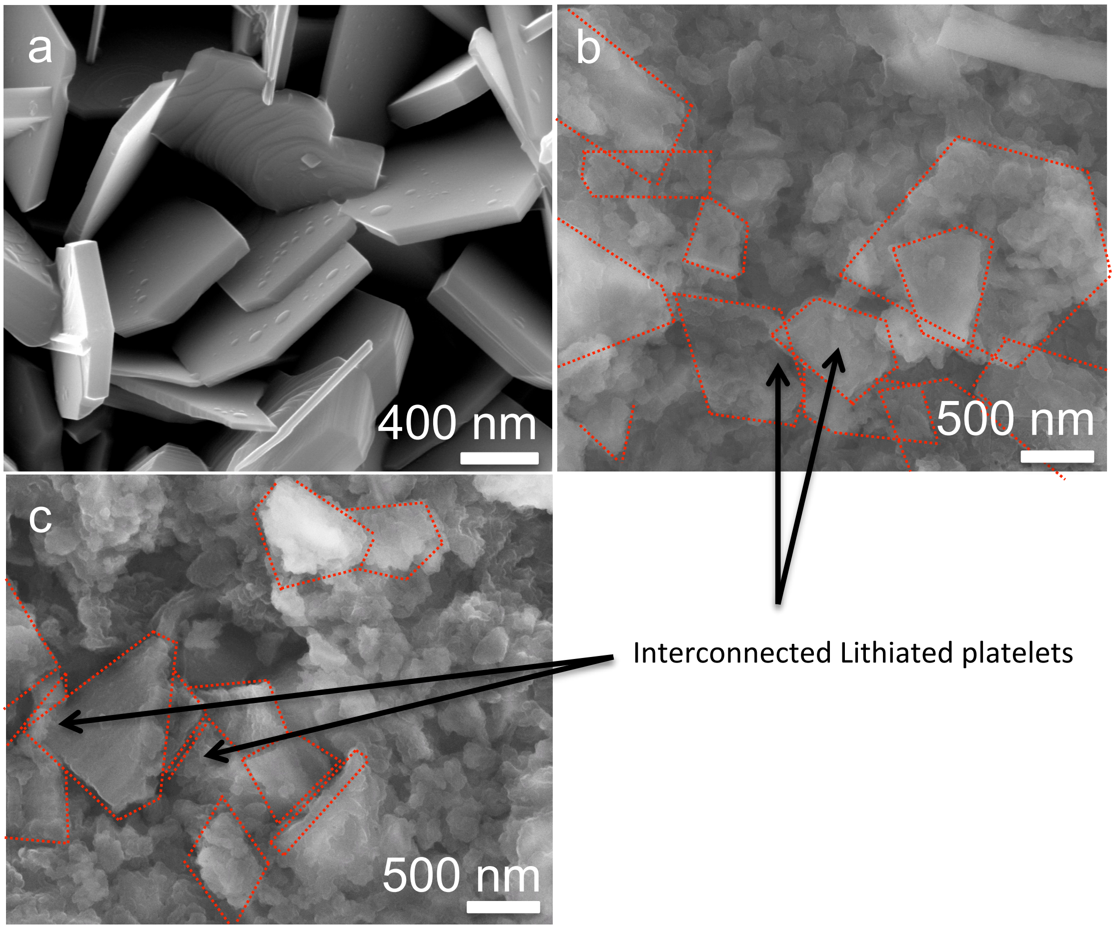

Figure S.IV a) SEM of interconnected MoO_3 nanoplatelets before lithiation, b) and c) Lithiated MoO_3 nanoplatelets

Details of the surface of MoO_3 nanoplatelets deposited by HFCVD can be seen in the scanning electron micrographs in Figure S.V. An abundance of screw dislocations formed by steps and terraces on the surface of each platelet suggest that the nanoplatelet morphology presents many sites for lithium intercalation and surface diffusion.

The growth characteristics and morphology of MoO_3 is governed by temperature at the filament and substrate, oxygen partial pressure, and deposition time. This particular shape of the crystal begins with the formation of 1D crystals (nanowires), then the nanowires extend in a second dimension forming platelets. If the temperature at the substrate exceeds a limit of around 500 ºC or the reaction time is extended, the nanoplatelets become thicker and ultimately this leads to formation of a film formed by cubic crystals. Typically, nanowire arrays have a blue color, while nanoplatelet networks are white to light grey, and the films are dark grey in color.

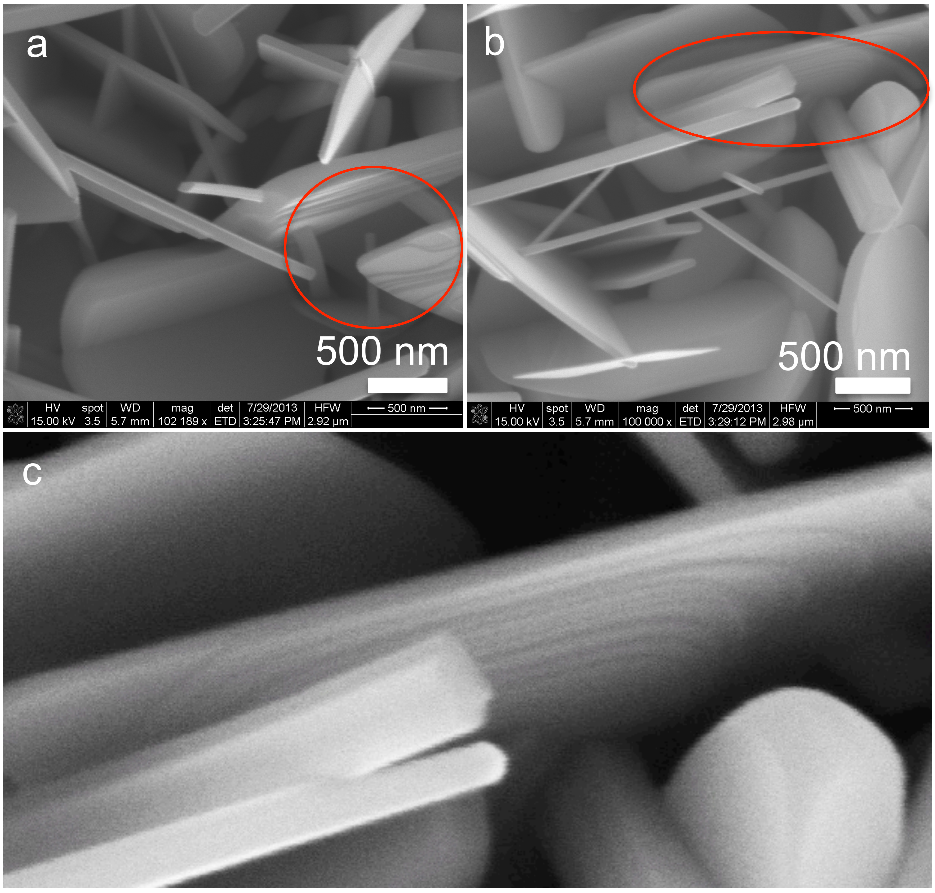

Figure S.V Edge and screw dislocations in MoO_3 nanoplatelets.

Discharge/charge plot for a MoO_3 electrode using a current of 100 mA g^{-1} is presented in Figure S.VI for the cycles # 1, 10, 20, 30, 50, and 100. These clearly shows a small plateau around 1.5V. This result is consistent with the cathodic peak present in the cyclic voltammogram of figure 2a of the main manuscript.

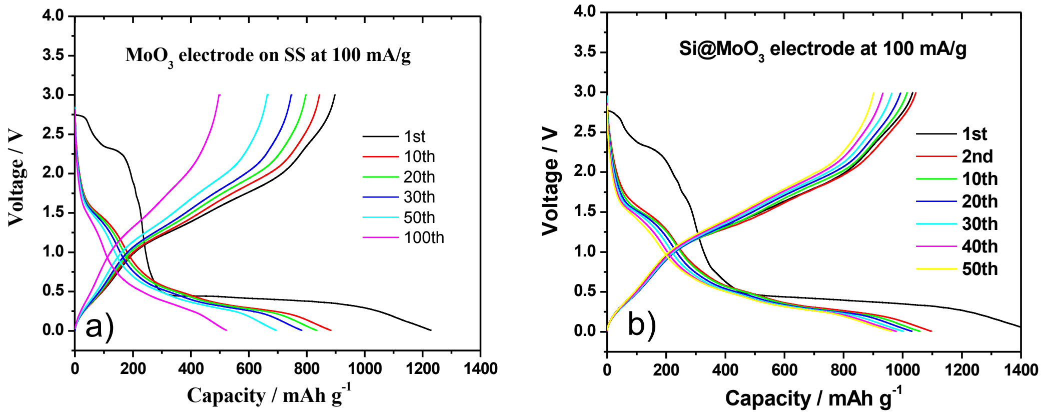

Figure S.VI The charge-discharge curves of electrodes at a current density of 100 mA/g. a) MoO_3 and b) Si@MoO_3

Bare silicon nanoparticles were studied electrochemically to assess the compatibility with MoO_3. For that purpose, silicon nanoparticles obtained by the procedure described in the method section were ultrasonically sprayed on a stainless steel coin-type substrate. Figure S.VII shows the cyclic voltammogram of a negative electrode only with silicon nanoparticles. A cathodic peak is present at a voltage of around 0.37 V vs. Li/Li^+, corresponding to the lithiation process and formation of Si-Li alloy. As stated in the manuscript, the majority of the capacity gain in the MoO_3-based anodes of this study occurs at a potential of 0.2 V vs. Li/Li^+ that is closer or below to the lithiation potential of silicon. As the lithiation potentials for both materials are so close, MoO_3 is a great host for silicon because a composite half-cell could operate at working voltages where MoO_3 won't get deeply discharged, and the voltage difference between the positive and negative electrodes can be safely and stably maximized in a wide range of the capacity.

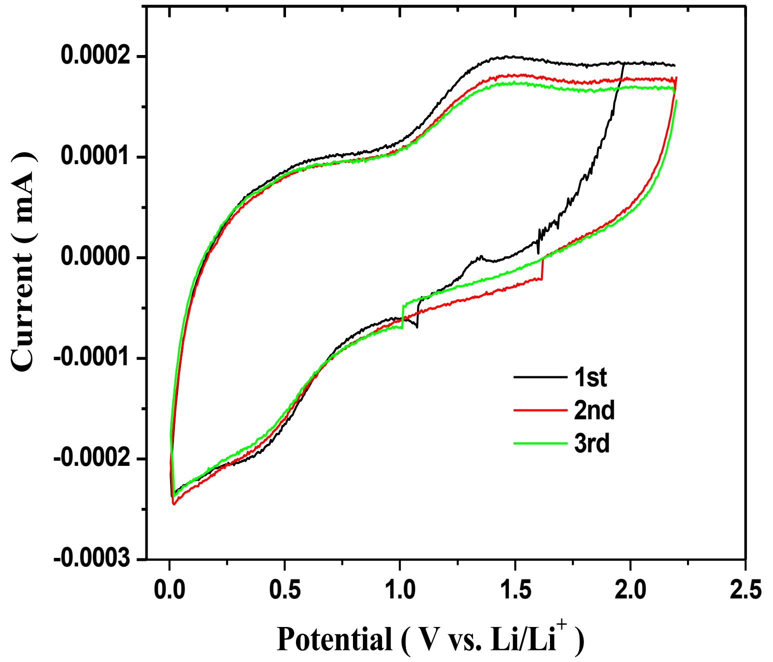

Fig S.VII. Cyclic voltammetry of pure silicon at the voltage range of 2.2 V-0.005 V with a scan speed of 5mV/s.

A comparison between two silicon loadings on MoO_3 electrodes is presented in Figure S. VIII. It is observed that while the initial capacity is large in the sample having 4 wt% Si (over 3000 mAh g^{-1}) the coulombic efficiency is very low as seen from the capacity of the second cycle.

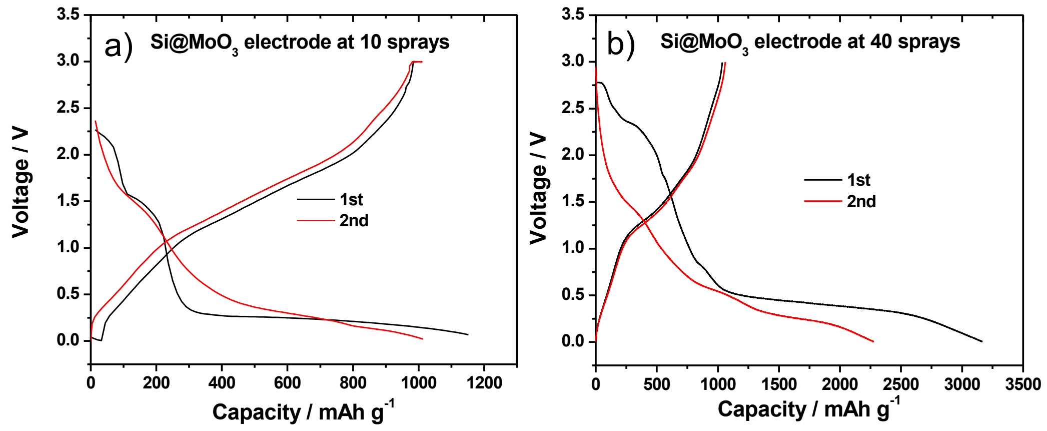

Fig S.VIII. Charge discharge curves for Si@MoO_3 electrodes with a) 1.5 wt% Si and b) 4 wt% Si

Figure S. IX shows the method used to spray silicon nanoparticles onto the substrates and also the procedure that was performed to estimate the loading of Silicon nanoparticles on the substrates based on the number of passes that each substrate was subjected to under the ultrasonic spray nozzle.

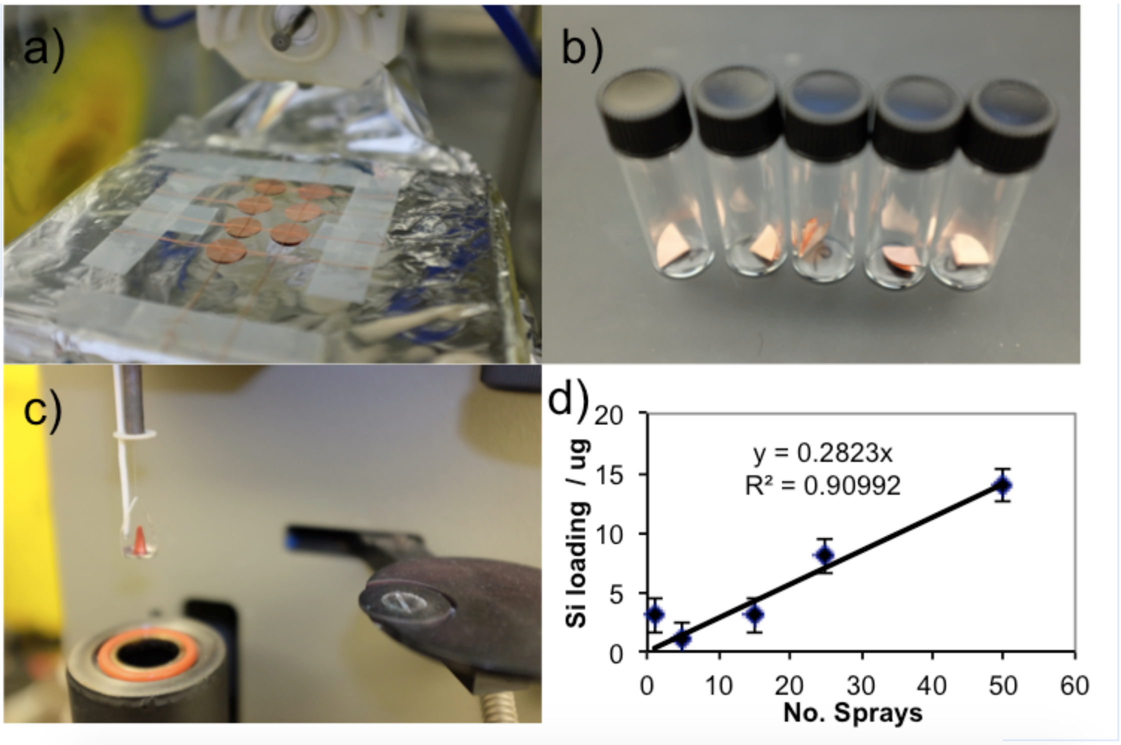

Fig S. IX. Method to estimate Silicon loading on substrates a) Copper Substrates being sprayed with silicon solution, b) Copper substrates with increasing number of passes under ultrasonic spray being transported to microbalance, c) Si-loaded copper electrode being weighed on a microbalance (chamber before being closed for measurement) d) Calibration curve
